# Enhancing clinical genomic accuracy with panelGC: a novel metric and tool for quantifying and monitoring GC biases in hybridization capture panel sequencing

**DOI:** 10.1093/bib/bbae442

**Published:** 2024-09-10

**Authors:** Xuanjin Cheng, Murathan T Goktas, Laura M Williamson, Martin Krzywinski, David T Mulder, Lucas Swanson, Jill Slind, Jelena Sihvonen, Cynthia R Chow, Amy Carr, Ian Bosdet, Tracy Tucker, Sean Young, Richard Moore, Karen L Mungall, Stephen Yip, Steven J M Jones

**Affiliations:** Canada’s Michael Smith Genome Sciences Centre, 570 W 7th Ave, Vancouver, British Columbia, V5Z 4S6, Canada; Canada’s Michael Smith Genome Sciences Centre, 570 W 7th Ave, Vancouver, British Columbia, V5Z 4S6, Canada; Canada’s Michael Smith Genome Sciences Centre, 570 W 7th Ave, Vancouver, British Columbia, V5Z 4S6, Canada; Canada’s Michael Smith Genome Sciences Centre, 570 W 7th Ave, Vancouver, British Columbia, V5Z 4S6, Canada; Canada’s Michael Smith Genome Sciences Centre, 570 W 7th Ave, Vancouver, British Columbia, V5Z 4S6, Canada; Canada’s Michael Smith Genome Sciences Centre, 570 W 7th Ave, Vancouver, British Columbia, V5Z 4S6, Canada; Canada’s Michael Smith Genome Sciences Centre, 570 W 7th Ave, Vancouver, British Columbia, V5Z 4S6, Canada; Canada’s Michael Smith Genome Sciences Centre, 570 W 7th Ave, Vancouver, British Columbia, V5Z 4S6, Canada; Cancer Genetics and Genomics Laboratory at BC Cancer Agency, 600 W 10th Ave #3305, Vancouver, British Columbia, V5Z 4E6, Canada; Cancer Genetics and Genomics Laboratory at BC Cancer Agency, 600 W 10th Ave #3305, Vancouver, British Columbia, V5Z 4E6, Canada; Cancer Genetics and Genomics Laboratory at BC Cancer Agency, 600 W 10th Ave #3305, Vancouver, British Columbia, V5Z 4E6, Canada; Cancer Genetics and Genomics Laboratory at BC Cancer Agency, 600 W 10th Ave #3305, Vancouver, British Columbia, V5Z 4E6, Canada; Cancer Genetics and Genomics Laboratory at BC Cancer Agency, 600 W 10th Ave #3305, Vancouver, British Columbia, V5Z 4E6, Canada; Canada’s Michael Smith Genome Sciences Centre, 570 W 7th Ave, Vancouver, British Columbia, V5Z 4S6, Canada; Canada’s Michael Smith Genome Sciences Centre, 570 W 7th Ave, Vancouver, British Columbia, V5Z 4S6, Canada; Canada’s Michael Smith Genome Sciences Centre, 570 W 7th Ave, Vancouver, British Columbia, V5Z 4S6, Canada; Canada’s Michael Smith Genome Sciences Centre, 570 W 7th Ave, Vancouver, British Columbia, V5Z 4S6, Canada

**Keywords:** targeted sequencing, GC bias, copy number variation, fragment abundance, hybridization capture panel sequencing, clinical genomics

## Abstract

Accurate assessment of fragment abundance within a genome is crucial in clinical genomics applications such as the analysis of copy number variation (CNV). However, this task is often hindered by biased coverage in regions with varying guanine–cytosine (GC) content. These biases are particularly exacerbated in hybridization capture sequencing due to GC effects on probe hybridization and polymerase chain reaction (PCR) amplification efficiency. Such GC content–associated variations can exert a negative impact on the fidelity of CNV calling within hybridization capture panels. In this report, we present panelGC, a novel metric, to quantify and monitor GC biases in hybridization capture sequencing data. We establish the efficacy of panelGC, demonstrating its proficiency in identifying and flagging potential procedural anomalies, even in situations where instrument and experimental monitoring data may not be readily accessible. Validation using real-world datasets demonstrates that panelGC enhances the quality control and reliability of hybridization capture panel sequencing.

## Introduction

Advancements in clinical genomics with next-generation sequencing (NGS) have revolutionized our understanding and diagnosis of genetic disorders. Central to this progress is accurate assessment of fragment abundance within a genome, enabling identification of copy number variations (CNVs). However, this pursuit is often hindered by regional guanine–cytosine (GC) content variation that affects DNA binding temperature, secondary structure formation, and polymerase chain reaction (PCR) efficiency ultimately leading to biased read coverage across a genome [1, 2]. Notably, these biases are exacerbated in hybridization capture sequencing, an economically efficient molecular tool widely employed to investigate Mendelian diseases and cancer [3–6]. Because this technique employs hybridization probes with varying GC content and thermocycler amplification steps, GC biases may emerge in various stages including library preparation, capture kits, different sequencers, and mapping processes. Instrument and reagent abnormalities can result in spurious calls and reduced sensitivity, even when GC bias correction algorithms are applied [2, 7].

While the GC effect is typically unimodal in whole genome sequencing (WGS) data [1, 2], it is rarely reported in the context of targeted sequencing. Existing tools, primarily developed for WGS data, fall short in several key areas relevant to targeted sequencing in clinical settings (Table 1): some tools only assess raw data from the sequencer without considering genomic context [8]; many visualize relationships between GC content and read depth without providing quantification in the form of a summary metric [9, 10]; and several tools estimate adenine–thymine (AT) and GC dropout without quantifying the degree of bias [11, 12]. Because these tools do not quantify the extent of GC bias or automatically distinguish abnormal from normal variations, assessment of GC bias patterns can require time-consuming and subjective qualitative manual interpretation. Importantly, existing tools have not been specifically developed for or extensively tested on capture sequencing data, and they do not address systemic issues at the batch level. Finally, current tools typically process samples in series rather than in parallel, which limits efficiency; the ability to process multiple samples simultaneously would greatly benefit clinical workflows where rapid and accurate detection of abnormalities are essential.

**Table 1:** Comparison of GC bias quality control tools in hybridization capture sequencing.

**Feature**	**panelGC**	**fastQC**	**Picard**	**deepTools**	**Qualimap**
Quantifiable bias score	Y	N	Y	N	N
Quantification—degree of bias	Y	N	Y	N	N
Quantification—direction of bias (AT versus GC)	Y	N	Partial	N	N
Automatic bias classification	Y	N	N	N	N
Developed for targeted sequencing data	Y	N	N	N	N
Visualization on a single sample	Y	Y	Y	Y	Y
Visualization on multiple samples	Y	N	N	N	N
Visualization on chronological trend	Y	N	N	N	N
Alignment awareness	Y	N	Y	Y	Y
Multisample input	Y	N	N	N	N
Multisample parallelization	Y	N	N	N	N

**Figure 1:**
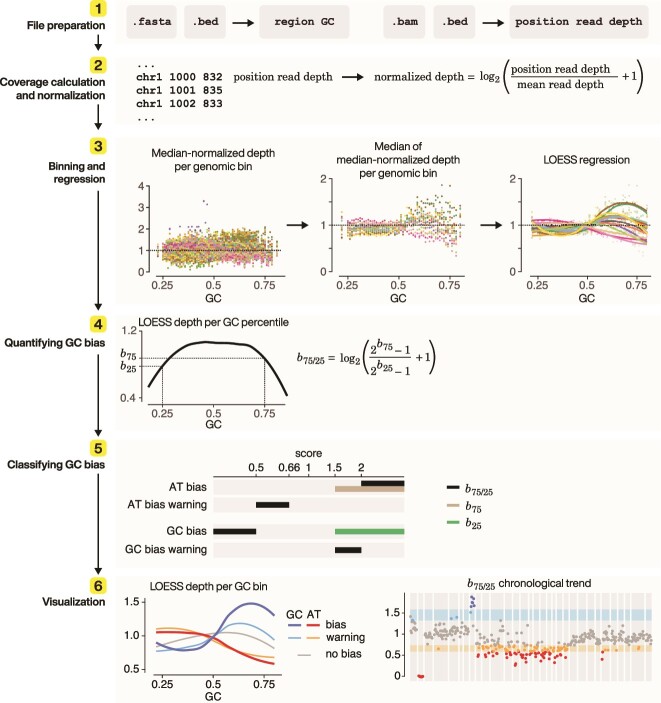
Schematic representation of panelGC algorithm. Step 1: Prepare inputs, including genome reference in FASTA format, read alignment in binary alignment map (BAM), and probe or genomic bins in BED format. Step 2: Intersect alignment files with the probes bed file (or genomic bin bed file) and compute per-position read depth. Normalize per-position read depth against the mean read depth, and log2 transform. Step 3: For each sample, calculate the median normalized depth across positions for each genomic bin, and determine the median of the medians for genomic bins sharing the same GC content. Perform LOESS regression on this median of the median. Each sample is represented with a distinct curve or dots. Step 4: Obtain LOESS read depth at 25% AT-anchor and 75% GC-anchor, corresponding to absolute fold changes *b*_25_ and *b*_75_. Calculate the relative fold change *b*_75/25_. Step 5: Classify GC biases based on the values of *b*_25_, *b*_75_, and *b*_75/25_. The *x*-axis represents the bias score, indicating bias magnitude. Lower scores indicate a greater extent of bias toward AT pairs, while higher scores indicate a greater extent of bias towards GC pairs. Step 6: Generate GC bias profile curves. Different patterns represent AT bias, AT bias warning, GC bias warning, GC bias, and no bias. LOESS, locally weighted scatterplot smoothing.

To address these challenges, we introduce panelGC, a standardized novel metric, to quantify, visualize, and monitor GC biases in hybridization capture sequencing data. We establish the efficacy of panelGC, demonstrating its proficiency in not only detecting GC biases but also flagging potential procedural irregularities, even in the absence of available instrumental quality control (QC) monitoring. We present the algorithm (Fig. 1) and implementation (https://github.com/easygsea/panelGC.git) of panelGC, and a comprehensive evaluation of its effectiveness, underscoring its potential to advance the accuracy and reliability of QC monitoring and variant calling within clinical genomics settings.

## Material and methods

### Clinical panels and data source

Our current clinical services (https://www.bcgsc.ca/services/clinical-services, accessed April 2, 2024) employ a batching process for genetic testing and include two specialized panels that we evaluate in this report for GC bias:

- Hereditary Cancer panel: Germline testing that detects small mutations in 82 genes and CNVs in 83 genes associated with inherited cancer predisposition syndromes.
- Myeloid panel: Identification of small mutations in 51 genes, FLT3 internal tandem duplication (FLT3/ITD), and KMT2A partial tandem duplication (KMT2A-PTD) for myeloid disorders, including acute myeloid leukemia, myelodysplastic syndrome, and myeloproliferative neoplasm.

Typically, each batch processes 20–30 samples for the Hereditary panel and around 8–10 for the Myeloid panel. A longitudinal retrospective dataset from clinical production batches was collected for this study (Supplementary Table 1). This dataset specifically included batches exhibiting signs of abnormalities linked to deviations in wet-lab procedures, with a noteworthy emphasis on instrumental abnormalities related to thermocyclers.

### Probe or genomic bins

Assessment of GC content effect involves dividing the reference sequence into specific-sized bins or windows [1, 9, 13]. In capture sequencing, probes are typically 100–120 bp in length, naturally functioning as scanned genomic bins, albeit with overlaps. In this study, we utilized 120 bp probe regions. The ideal size for the scanning windows on the sequenced reference genome can be determined by each individual group. Bins are submitted in the browser extensible data (BED) format (Fig. 1, Step 1).

### Coverage calculation and normalization

Normalization is performed to generate a comparable metric across libraries and sequencing batches to adjust raw data for factors like varying library sizes [14] that prevent direct comparison of coverage enabling meaningful coverage comparison. We first used BEDTools [15] to calculate the per-nucleotide read depth (Fig. 1, Step 2). Subsequently, we implemented a normalization strategy similar to ‘CollectGcBiasMetrics’ within the Genome Analysis Toolkit (GATK) [13]. The normalized depth per position ($Normalized\_ Depth$) is calculated by dividing the number of reads per position ($Position\_ Read\_ Depth$) by the mean number of mapped reads across all probed positions ($Mean\_ Read\_ Depth$), which is then incremented by 1 and subjected to a logarithmic transformation with a base of 2:


$$ Normalized\_ Depth={\mathit{\log}}_2\left(\frac{Position\_ Read\_ Depth}{Mean\_ Read\_ Depth}+1\right) $$

A value of 1 represents mean coverage, while a value below 1 indicates coverage lower than the mean, and a value exceeding 1 indicates coverage higher than the mean.

### Binning and regression

The median normalized depth of each query genomic bin is first calculated (Fig. 1, Step 3 left panel). Subsequently, bins are grouped into GC percentiles, and the median of these median normalized depths for bins within the same GC percentile is calculated to provide outlier resistance (Fig. 1, Step 3 middle panel). A GC bias regression curve is calculated using the locally estimated scatterplot smoothing (loess) regression, with the “loess” package in R [16], to correlate these depths with GC content (Fig. 1, Step 3 right panel). The regressed depth is henceforth denoted as LOESS depth.

### Quantifying GC biases

Variation in experimental conditions can lead to imbalances in read depth in AT- or GC-rich regions. We propose two methods to quantify GC biases using the regression curve obtained above:

- GC-percentile-anchored fold change: The LOESS depth, as derived above, functions as a measure of fold change in comparison to the mean coverage. We propose using two GC percentiles to establish anchors for AT- and GC-rich regions. In this study, we opted for 25% and 75% (refer to Supplementary Fig. 1 for probe GC content distribution). GC content between 25% and 75% is often considered optimal for genomic applications, making these thresholds effective markers for AT-rich and GC-rich regions ​ [1, 17–20]. Extreme GC content values often correspond to low coverage regions and are susceptible to noise, and anchors beyond the 40%–60% range are typically considered GC-neutral and thus less informative. Our empirical observations show that 25% and 75% GC content provide stable performance and is resistant to random dropouts while maintaining sensitivity to deviations from normal. Given variations in panel design and workflows, we recommend selecting anchor combinations based on the GC content distribution of the probes (or genomic bins) and/or empirical testing to identify the optimal anchor pair. Once anchors are selected, we calculate the relative fold change between the predicted normalized depth at 25% GC (AT-anchor) and that at 75% GC (GC-anchor), which we term *b*_75/25_, as the GC bias score. Absolute fold changes at the AT-anchor (*b*_25_, LOESS depth at 25% GC) and the GC-anchor (*b*_75_, LOESS depth at 75% GC) can be used in conjunction with the relative fold change *b*_75/25_ score as additional support (see Results).
  $$ {b}_{75/25}={\mathit{\log}}_2\left(\frac{2^{b_{75}}-1}{2^{b_{25}}-1}+1\right) $$
- Area under the curve: A second approach is to calculate the difference in area under the curve for GC ≥50% against that for GC <50%. This calculation can be performed by approximating the area using a calculus-like method, which involves summing the predicted depth at each GC percentile.

The first approach provides fold change values, allowing thresholding at interpretable 1.5- or 2-fold levels [21]. Conversely, the second approach necessitates empirical derivation of threshold values. In our validation process, we opted for the first approach, as it is sufficiently sensitive to detect instrumental abnormalities, as elaborated upon in the Results section.

### Thresholds for classifying GC biases

To identify and classify samples affected by GC biases, we apply specific fold change criteria. A relative fold change threshold (relative_fold_change_threshold) is used for *b*_75/25_, while an absolute fold change threshold (absolute_fold_change_threshold) is applied for *b*_75_ and *b*_25_. The thresholds are defined as follows:

GC bias failure:

*b*_75/25_ ≥ 1.584963 [or log_2_(2 + 1)]: LOESS depth at 75% GC is at least 2 times higher than at 25% GC, and/or

*b*_75_ ≥ 1.321928 [or log_2_(1.5 + 1)]: LOESS depth at 75% GC is at least 1.5 times higher than the mean coverage.

AT bias failure:

*b*_75/25_ ≤ 0.584962 [or log_2_(1/2 + 1)]: LOESS depth at 25% GC is at least 2 times higher than at 75% GC, and/or

*b*_25_ ≥ 1.321928 [or log_2_(1.5 + 1)]: LOESS depth at 25% GC is at least 1.5 times higher than the mean coverage.

GC bias warning:

1.321928 [or log_2_(1.5 + 1)] ≤ *b*_75/25_ < 1.584963 [or log_2_(2 + 1)]: LOESS depth at 75% GC is 1.5 times higher than at 25% GC.

AT bias warning:

0.584962 [or log_2_(1/2 + 1)] < *b*_75/25_ ≤ 0.736965 [or log_2_(1/1.5 + 1)]: LOESS depth at 25% GC is 1.5 times higher than at 75% GC.

### Visualization

**Figure 2:**
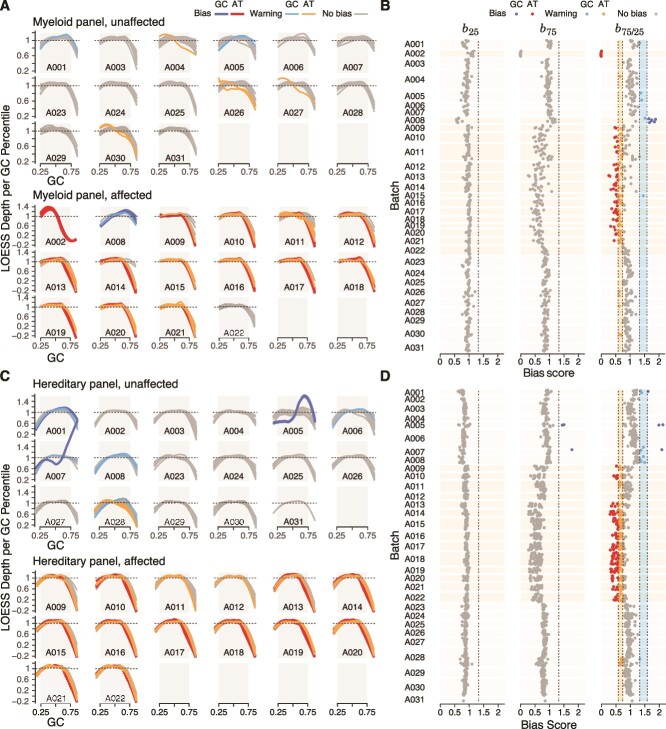
panelGC validation results. (A) GC profile plots depict samples processed with the Myeloid panel in normal batches (top) and batches affected by experimental irregularities (bottom). (B) Longitudinal visualization presents bias scores of samples processed with the Myeloid panel over time. (C) GC profile plots depict samples processed with the Hereditary panel in normal batches (top) and batches affected by experimental irregularities (bottom). (D) Longitudinal visualization presents bias scores of samples processed with the Hereditary panel over time.

In conjunction with the metric, we concurrently developed two visualization tools that augment its analytical capabilities. One visualization plots the LOESS regression curves colored according to thresholds, and the other shows *b*_75/25_, *b*_75_, and *b*_25_ scores colored according to thresholds and plotted over time (Fig. 1, Step 6). Together, they enable both identification of abnormal samples and tracking of trends over time (Fig. 2).

### Implementation

We implemented panelGC with Nextflow [22]. Essential packages, including bedtools [15], r-base [23], and dependent R packages, are executed from Singularity containers [24]. The source code is available at GitHub (https://github.com/easygsea/panelGC.git).

### Comparative analysis of GC bias quality control tools

panelGC was compared against Picard [11], deepTools [10], and Qualimap [9], using three categories of datasets:

- Clinical hybridization capture sequencing datasets compiled in Supplementary Table 1.
- Simulated panel datasets from published sources: Two published datasets with normal GC bias variations, SRX040660 and SRX040661 [1], were intersected with the Integrated DNA Technologies (IDT) xGen Inherited Diseases Hyb Panel [25] probes to simulate targeted panel data.
- Simulated panel datasets with controlled GC and AT biases: A total of 100 autosomal genes were randomly selected from the IDT xGen Inherited Diseases Hyb Panel [25]. The GC content for each probe was calculated. Paired-end 150 bp sequencing reads were simulated with varying depths, specifically targeting AT- and GC-rich regions. This included datasets with minimal bias, moderate bias, and high bias to assess the performance of each tool under different conditions.

## Results

### Validation and detection of abnormalities in experimental procedures

By examining the panelGC outputs across production batches, we identified abnormal samples and the temporal scope of the anomalies.

- Abnormal sample detection: The metric enabled identification of samples affected by deviations in wet-lab procedures, uncovering subtle irregularities that had gone unnoticed (Fig. 2). In batch A002, zero reads and widespread AT biases were observed in samples processed with the Myeloid panel, later linked to an over-heated wash buffer leading to overly stringent capture. Similarly, in batch A008, we observed GC biases in samples processed with the Myeloid panel attributed to heightened off-target read alignments and elevated duplication rates. Furthermore, an investigation of batches A009 to A022 highlighted the impact from a thermocycler as a major source of AT biases in multiple samples, confirmed to stem from flaws in multiblock thermal cycling, undetected in regular maintenance assessment.
- Trend and temporal analysis: One of the most notable findings in our longitudinal dataset was the precise identification of the moment when problems began to emerge. Illustratively, in batches A009–A022 impacted by the thermocycler, systematic adjustments were made to experimental parameters. Shifting samples from the Myeloid panel toward the center of the plate in A022 appeared to alleviate some biases, but it was the replacement of the old thermocycler with a new one in A023 that conclusively resolved biases across all panels (Fig. 2). This temporal analysis enabled us to isolate the root cause to the thermocycler and precisely pinpoint effective corrective actions.

The synergistic combination of panelGC and its visualizations enable us to pinpoint, interpret, and respond to abnormalities in the clinical NGS production process. This integrated approach elevated our capacity to make informed decisions and implement proactive measures, ensuring the integrity and reliability of our clinical production processes.

### Application in real-world clinical monitoring

**Figure 3:**
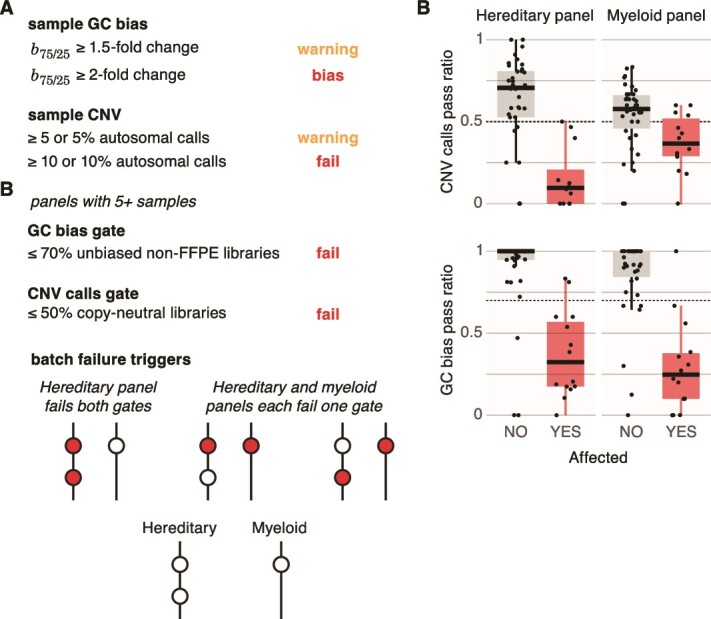
Workflow for integrated panelGC and CNV monitoring. (A) The diagram outlines the logic for identifying GC bias or abnormal copy event count in a sample. (B) Empirical data demonstrate that a minimum of 70% of libraries exhibiting no GC bias and 50% showing copy neutrality can distinguish batches affected by experimental irregularities from those that are not. (C) The logistics diagram outlines the process for detecting systemic GC bias in a sequencing batch, triggering an investigation. CNV, copy number variation; FFPE, formalin-fixed paraffin-embedded; QC, quality control.

**Figure 4:**
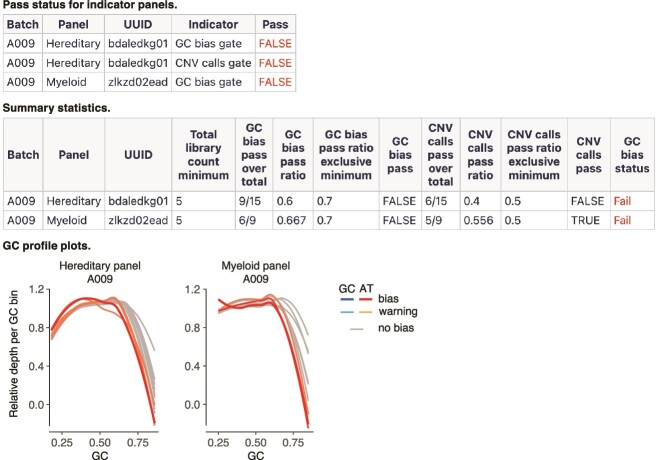
Example output of integrated panelGC and CNV calls monitoring. Total sample count minimum: The minimum number of samples within a panel for gate evaluation. GC bias pass over total: number of samples showing no bias over the total number of samples within the panel. GC bias pass ratio: percentage of samples showing no bias within the panel. GC bias pass ratio exclusive minimum: the minimum percentage of samples showing no bias to deem a panel passing the GC bias gate. GC bias pass: Boolean indicating whether the panel passes the GC bias gate. CNV calls pass over total: number of copy-neutral samples over the total number of samples within the panel. CNV calls pass ratio: percentage of copy-neutral samples within the panel. CNV calls pass ratio exclusive minimum: The minimum percentage of copy-neutral samples to deem a panel passing the CNV calls gate. CNV calls pass: Boolean indicating whether the panel passes the CNV calls gate. GC bias status: “fail” signifies a GC bias issue in the panel, while “pass” indicates no obvious GC bias issue. CNV, copy number variation; UUID, Universal Unique Identifier.

In our pursuit of a comprehensive solution for clinical monitoring, we extended the practical application of panelGC beyond sample-specific issue detection into the realm of systemic panel and batch-level abnormalities. To this end, we integrated panelGC with CNV calls (Fig. 3). Rather than focusing on failure rates, we set thresholds for success rates:

Sample normality definition (Fig. 3A):

- GC bias: Fails at *b*_75/25_ ≥2-fold change; warns at *b*_75/25_ ≥1.5-fold change. A sample is considered free from GC bias if below these thresholds.
- Number of CNV calls: Fails with ≥10 or 10% nondiploid autosomal calls; warns at ≥5 or 5%. A sample is considered copy neutral if only <5 and <5% of its autosomal calls are nondiploid.
- Empirical threshold determination for panels (Fig. 3B): Our empirical analysis, spanning the past year of production, identified a minimum panel size of five samples. In normal batches, over 70% of samples should be free of GC bias, and >50% should be copy neutral.
- Batch failure logic (Fig. 3C): Building upon the panel failure status established above, we applied the logic outlined in Fig. 3C. Briefly, a panel fails the GC bias gate if over 30% of its samples exhibit biases. Additionally, the Hereditary panel, covering CNV analysis for 83 genes, also fails if over 50% of its samples are noncopy neutral. The Myeloid panel does not use a CNV calls gate since its CNV analysis targets FLT3 and KMT2A, genes relatively tolerant to GC biases. The GC bias and CNV calls gates are evaluated only for panels with five or more samples. A batch is considered failed only if both the Hereditary and Myeloid panels fail. This procedural step allowed us to effectively automate and standardize the protocol for batch-level issue monitoring and promptly feed back to the sequencing group (Fig. 4).

We also explored the application of the Kolmogorov–Smirnov (KS) test, a nonparametric test commonly used to determine if two samples are drawn from the same distribution [26]. This test, while valuable, posed specific challenges. Its accuracy depends on a well-defined panel of normal samples and careful thresholding of the *P*-value and KS distance. Issues arise when the reference sample is not representative, potentially leading to unreliable results. The test may also generate false positives in response to minor data variations or fail to detect significant discrepancies (Supplementary Results). Additionally, while the KS test can identify batch-level anomalies, it does not offer granularity at the level of individual libraries. We have shared these findings with the research community for consideration in their own dataset analysis.

### Evaluation of GC bias quality control tools

To evaluate the effectiveness of panelGC in detecting and quantifying GC bias in the context of capture sequencing, we compared its performance using default parameters against three widely used tools [9–11].

- Bias score comparisons: Picard [11] CollectGcBiasMetrics quantifies GC bias into scores, namely, AT_DROPOUT and GC_DROPOUT. We executed Picard (v3.1.1.20) on the clinical hybridization capture sequencing datasets and then compared panelGC’s *b*_75/25_ scores against Picard’s scores. Picard’s GC_DROPOUT scores often approached 0, limiting differentiation of libraries with abnormal increases in read depth in AT- or GC-rich regions (Supplementary Fig. 2A). Picard’s AT_DROPOUT scores provided a broader range but sometimes failed to distinguish between biased and nonbiased libraries (Supplementary Fig. 2B). In contrast, panelGC automatically classified and flagged abnormal biases, effectively distinguishing increased read depth in AT- and GC-rich regions (Supplementary Fig. 2). Correlation analysis showed a weak negative correlation between panelGC’s *b*_75/25_ and Picard’s GC_DROPOUT metrics and a moderate positive correlation with Picard’s AT_DROPOUT metrics (Supplementary Fig. 2).
- Visual comparisons: deepTools [10] and Qualimap [9] offer visualizations but no quantifiable bias scores. We ran deepTools (v3.5.5), Qualimap (v2.3), and Picard CollectGcBiasMetrics (v3.1.1) on two clinical capture sequencing cell line samples (one from a normal batch and one from a problem batch) and the simulated panel datasets. deepTools tended to regress higher read depth toward GC-rich regions (Supplementary Figs 3A and 4A). Qualimap and Picard regressed all samples toward a unimodal pattern (Supplementary Figs 3B and C and 4B and C), limiting differentiation between normal and biased samples. panelGC successfully identified unimodal patterns for SRX040660 and SRX040661 on the simulated hybridization panel, consistent with the original publication [1], and flagged abnormalities in both real clinical and controlled simulation settings, reducing the need for manual interpretation (Supplementary Fig. 3D).
- Processing speed: panelGC demonstrated improved processing speed over current tools by parallelizing the analysis of multiple samples (Table 2). This enhanced speed, along with the elimination of manual interpretation, is particularly beneficial for clinical workflows where turnaround time is critical.
- Applicability across varying sequencing depths: panelGC consistently detected and quantified GC biases effectively at both low and high sequencing depths in the benchmarked datasets, ranging from 5X to over 1000X.

**Table 2:** Tool computational efficiency comparison.

**Dataset**	**Sample size**	**Typical sequencing depth**	**Panel space (bp)**	**panelGC**	**Picard**	**deepTools**	**Qualimap**
Hereditary panel capture sequencing	607	>1000X	0.55 million	44.3 (−^a^)	170.8 (16.0)	–	–
Myeloid panel capture sequencing	283	>1000X	0.26 million	37.7 (−^a^)	152.8 (8.7)	–	–
Cell line capture sequencing	2	>1000X	0.3 million	90.5 (−^a^)	163.0 (8.5)	199.5 (10.6)	149.0 (7.1)
Simulated panel data from SRX040660 SRX040661	2	5X	11 million	99.5 (−^a^)	133.5 (14.8)	157.0 (1.4)	48.0 (1.4)
Simulated panel data with controlled GC and AT biases	5	10X	0.2 million	8.1 (−^a^)	134.3 (2.3)	20.6 (4.3)	23.4 (0.5)

Average run time (seconds) is reported per sample. Numbers in parentheses denote standard deviation. bp, base pair.

a Average run time per sample is reported. Standard deviation is not applicable because samples are processed in parallel.

These comparisons demonstrate the consistency of panelGC with other tools and highlight cases where panelGC provides clearer insights into GC bias. panelGC excels in key areas relevant to clinical capture sequencing workflows: precise quantification of bias extent and direction at both low and high sequencing depths, automatic classification and detection of abnormalities, and enhanced processing speed. These benefits are particularly crucial in clinical settings, where the detection of abnormalities is essential and turnaround time is critical.

## Discussion

Our study highlights a critical distinction between WGS and targeted sequencing approaches in terms of GC bias patterns. Unlike a unimodal curve typically observed with WGS data [1, 2], hybridization capture panel sequencing datasets display a variety of curve patterns. This diversity, influenced by probe design and a smaller genomic footprint, often results in over-representation of high or low GC regions, complicating the accurate quantification and mitigation of GC biases.

Existing algorithms designed to correct GC bias during specific bioinformatic applications [27, 28] often fall short when confronted with instrument and reagent-induced abnormalities. These anomalies can introduce biases that post-sequencing normalization cannot fully correct, leading to spurious variant calls and reduced sensitivity [2, 7, 29]. Correction methods generally rely on certain assumptions, and when these assumptions are violated due to experimental issues, data distortion can occur. As Lander *et al*. [30] demonstrated, detecting bias is fundamental to developing robust correction strategies. Thus, ensuring datasets are free from experimental problems is vital before applying correction methods.

The use of a ‘median of the median’ approach in calculating the depth of each GC bin is a key aspect of the panelGC methodology. This strategy effectively mitigates the impact of outliers. By first calculating the median depth of each genomic bin and subsequently grouping them into GC percentiles, we take measures to prevent the undue influence of extreme values on the results.

We opted for a fold change–based approach to quantify GC biases, aiming to provide a meaningful indication of the impact of GC content on read depth. The absolute fold changes observed in regions rich in AT and GC content (as captured by *b_75_* and *b_25_*), as well as the relative fold change between these regions (as captured by *b_75/25_*), provide a straightforward measure of the magnitude of bias. While we considered statistical testing methods like the KS test, we found them to be overly sensitive to subtle variations and reliant on a well-defined panel of normal samples. The fold change approach emerged as a more practical and effective solution for detecting GC biases within the context of our specific application.

The integration of panelGC with the number of CNV calls and the establishment of thresholds for success rates at the panel and batch levels has improved monitoring in our clinical workflow. This integrated approach enables the automated detection of batch-level issues and provides timely feedback to the sequencing group. It showcases the potential for synergistic utilization of multiple metrics and data types in clinical genomics workflows to enhance quality control and assurance.

It is important to acknowledge the specific context in which panelGC has been evaluated. Our evaluation employed fresh germline samples with NGS readout. Formalin-fixed and paraffin-embedded (FFPE) samples present additional challenges and sources of bias that were not specifically addressed in this study. Therefore, caution and further research are recommended when applying panelGC to datasets derived from FFPE samples or non-NGS assays.

Lastly, while our study focuses on hybridization capture sequencing of human datasets, the methodology and principles underlying panelGC may extend to other types of targeted sequencing approaches and other organisms. The fundamental concept of assessing GC biases in a targeted region, characterized by probe- or region-based genomic bins, could potentially be adapted to other capture methods, such as amplicon-based sequencing. Furthermore, since panelGC uses alignment data as input, it could be applied to datasets from nonhuman sources, albeit with consideration of overall genomic GC profile and necessary testing and validation.

In summary, our results demonstrate a novel yet easily implemented metric adept at detecting GC biases at the sample, panel, and batch levels in hybridization capture methods. It demonstrates several key advantages in clinical workflows: quantification of bias extent and direction at both low and high sequencing depths, automatic classification and detection of abnormalities, and enhanced processing speed. The integration of panelGC with CNV results and the standardization of batch-wise monitoring protocols mark a significant step forward in the realm of real-world clinical monitoring, providing a useful tool for enhancing the integrity and quality of clinical hybridization capture sequencing.

**Key Points**

- GC bias quantification: We have introduced a precise measure of GC biases within hybridization capture panel sequencing data to facilitate quality control.
- Easy implementation: Open-source stand-alone tool for straightforward application.
- Comprehensive validation: The metric demonstrates a high correlation between panelGC scores and true GC biases when validated on real datasets.
- Clinical application: panelGC is used by a large sequencing center for monitoring clinical capture panel sequencing data to detect sample, panel, and batch-wise GC bias.

## Supplementary Material

panelGC_Supplementary_Data_R2V1_bbae442

## Data Availability

The panelGC source code is available at GitHub (https://github.com/easygsea/panelGC.git).
