# Supplementary material for: Enhancing clinical genomic accuracy with panelGC: a novel metric and tool for quantifying and monitoring GC biases in hybridization capture panel sequencing
Source: Brief Bioinform. 2024 Sep 10;25(5):bbae442. doi: 10.1093/bib/bbae442 (PMC11387050; doi:10.1093/bib/bbae442)
Supplement: panelGC_Supplementary_Data_R2V1_bbae442 [file panelgc_supplementary_data_r2v1_bbae442.pdf]

Supplementary Results

Kolmogorov-Smirnov (KS) test

We implemented the KS test with "ks.test" within the base "stats" package in R [1]. Parameters were kept at default settings unless specified otherwise in individual test descriptions.

Our approach involved comparing each batch against a "rolling normal reference", defined as the last five batches deemed normal. This method effectively accommodated shifts in library construction quality over time, ensuring the reference remained representative of the prevailing normal conditions.

To mitigate the risk of false positives due to multiple testing, we applied the Bonferroni correction method [2].

We set the following thresholds: an adjusted p-value < 0.05 and a KS distance ⩾ 0.7. These thresholds were determined empirically by analyzing affected batches in contrast to normal ones. The adjusted p-value threshold of <0.05 aligns with standard practices for statistical significance. Simultaneously, the KS distance threshold of ⩾ 0.7 was carefully chosen to distinguish between batches affected by anomalies and those classified as normal, reducing the likelihood of false positives.

Supplementary Table 2 show the results of the KS approach. Out of 62 tests, the KS approach correctly identified 59 biased outcomes. However, it failed to detect biases in 2 Myeloid batches and erroneously labelled 1 normal Myeloid batch as "Affected". This occurred despite our efforts in creating a rolling normal reference and carefully calibrating the adjusted p-value and KS distance thresholds.

REFERENCES

1. R Core Team. R: A language and environment for statistical computing. R Foundation for Statistical Computing, Vienna, Austria. URL https://www.R-project.org/. 2020;
2. Armstrong RA. When to use the Bonferroni correction. Ophthalmic Physiol. Opt. J. Br. Coll. Ophthalmic Opt. Optom. 2014; 34:502–508
3. Broad Institute. Picard toolkit. Broad Institute, GitHub repository 2019; https://broadinstitute.github.io/picard/
4. Ramírez F, Dündar F, Diehl S, et al. deepTools: a flexible platform for exploring deep-sequencing data. Nucleic Acids Res. 2014; 42:W187–W191
5. García-Alcalde F, Okonechnikov K, Carbonell J, et al. Qualimap: evaluating next-generation sequencing alignment data. Bioinformatics 2012; 28:2678–2679
6. Benjamini Y, Speed TP. Summarizing and correcting the GC content bias in high-throughput sequencing. Nucleic Acids Res. 2012; 40:e72

Supplementary Tables

Supplementary Table 1. Deidentified production batches. Affected: Presence of experimental irregularities, highlighted in red. Normal: No experimental irregularities.

Batch Number	Hereditary Panel Indication	Myeloid Panel Indication
A001	Normal	Normal
A002	Normal	Affected
A003	Normal	Normal
A004	Normal	Normal
A005	Normal	Normal
A006	Normal	Normal
A007	Normal	Normal
A008	Normal	Affected
A009	Affected	Affected
A010	Affected	Affected
A011	Affected	Affected
A012	Affected	Affected
A013	Affected	Affected
A014	Affected	Affected
A015	Affected	Affected
A016	Affected	Affected
A017	Affected	Affected
A018	Affected	Affected
A019	Affected	Affected
A020	Affected	Affected
A021	Affected	Affected
A022	Affected	Affected
A023	Normal	Normal
A024	Normal	Normal
A025	Normal	Normal
A026	Normal	Normal
A027	Normal	Normal
A028	Normal	Normal
A029	Normal	Normal
A030	Normal	Normal
A031	Normal	Normal

Supplementary Table 2. Kolmogorov-Smirnov (KS) test results. Affected: significant (adjusted p-value < 0.05 and KS distance ⩾ 0.7) deviation from the normal. Normal: No significant deviation from the normal. Instances incorrectly predicted, as compared to experimental records as documented in Table 1, are highlighted in red.

Batch Number	Hereditary Panel KS Result	Myeloid Panel KS Result
A001	Normal	Normal
A002	Normal	Affected
A003	Normal	Normal
A004	Normal	Normal
A005	Normal	Normal
A006	Normal	Normal
A007	Normal	Normal
A008	Normal	Normal
A009	Affected	Affected
A010	Affected	Affected
A011	Affected	Normal
A012	Affected	Affected
A013	Affected	Affected
A014	Affected	Affected
A015	Affected	Affected
A016	Affected	Affected
A017	Affected	Affected
A018	Affected	Affected
A019	Affected	Affected
A020	Affected	Affected
A021	Affected	Affected
A022	Affected	Affected
A023	Affected	Affected
A024	Normal	Normal
A025	Normal	Affected
A026	Normal	Normal
A027	Normal	Normal
A028	Normal	Normal
A029	Normal	Normal
A030	Normal	Normal
A031	Normal	Normal

Supplementary Figures

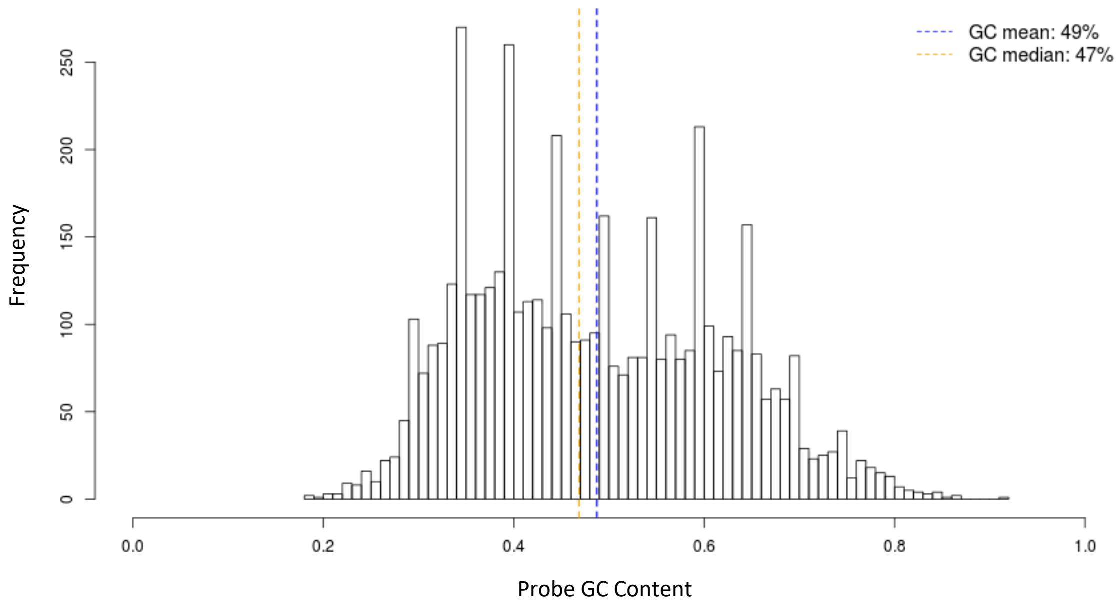

Supplementary Figure 1. Example distribution of probe GC content.

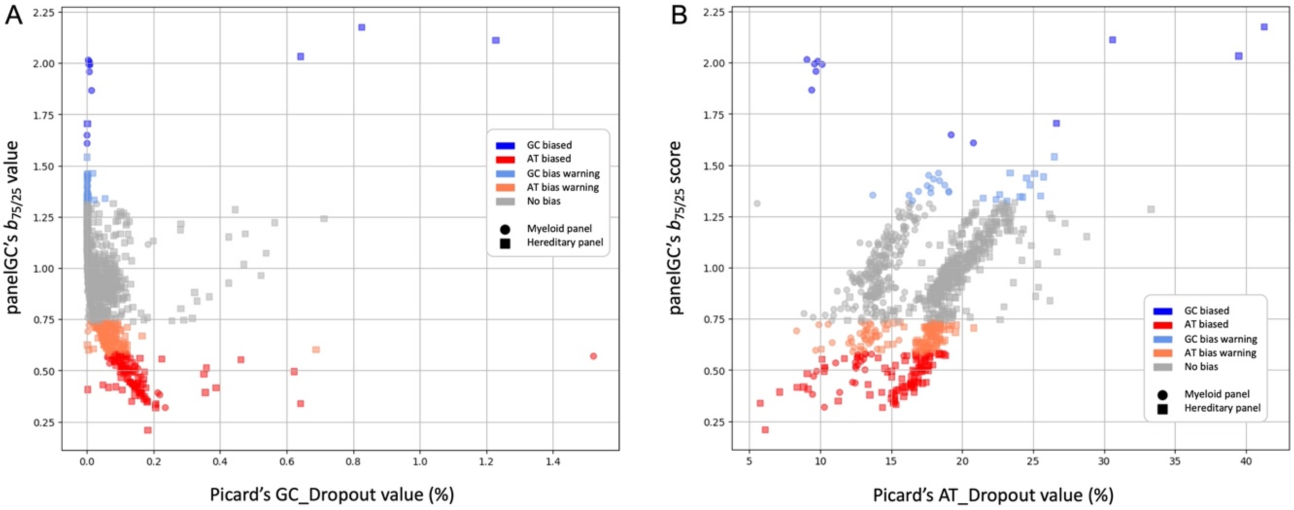

Supplementary Figure 2. Scatter plot comparing GC bias quantifications by Picard and panelGC. (A) Picard's GC_Dropout metric versus panelGC's $b_{75/25}$ metric: Myeloid panel r = -0.34, *p* = 1.66e-08; Hereditary panel r = -0.10, *p* = 0.01. **(A)** Picard's AT_Dropout metric versus panelGC's $b_{75/25}$ metric: Myeloid panel r = 0.41, *p* = 5.93e-12; Hereditary panel r = 0.78, *p* = 2.68e-129. r, Pearson correlation coefficient; *p*, *P*-value.

A

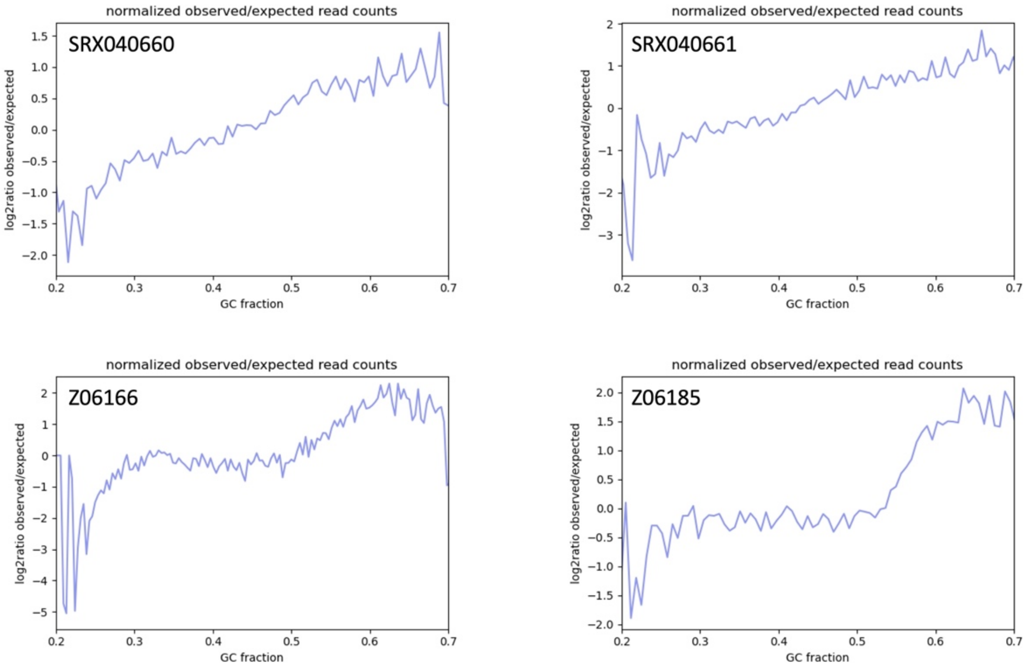
normalized observed/expected read counts
SRX040660
SRX040661
Z06166
Z06185
log2ratio observed/expected
GC fraction

B

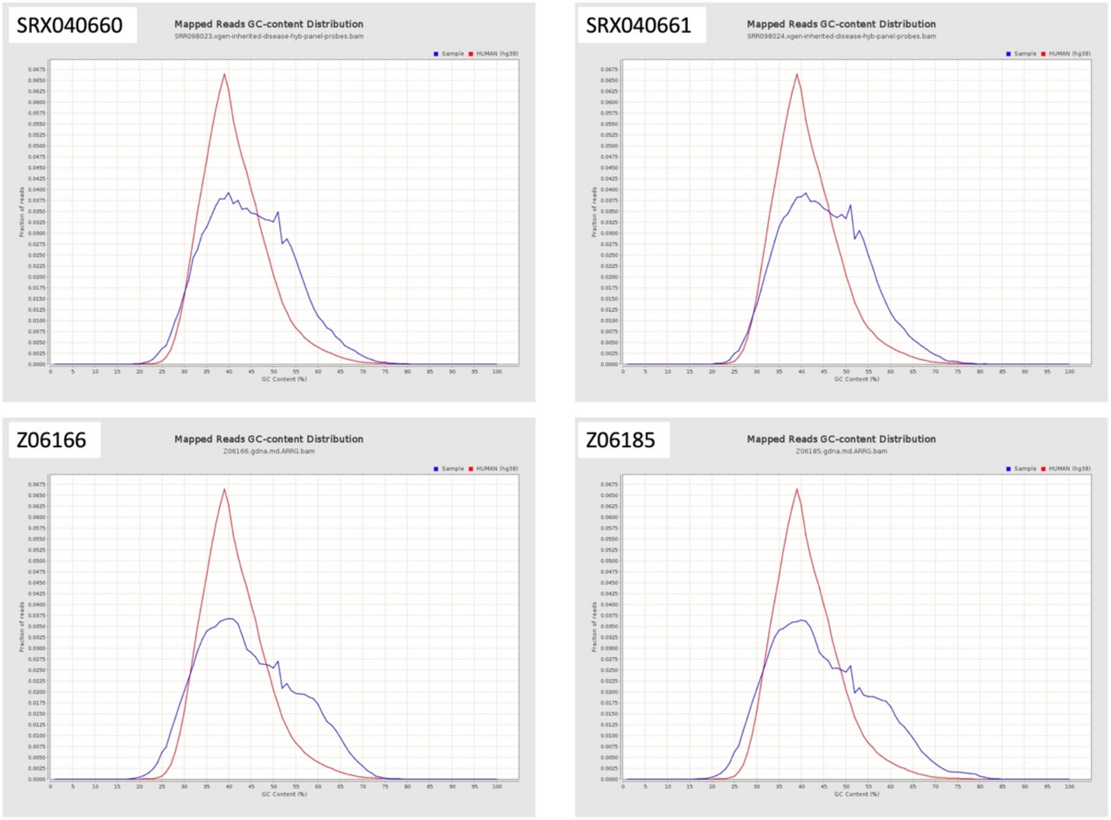
SRX040660
SRX040661
Z06166
Z06185
Mapped Reads GC-content Distribution

C

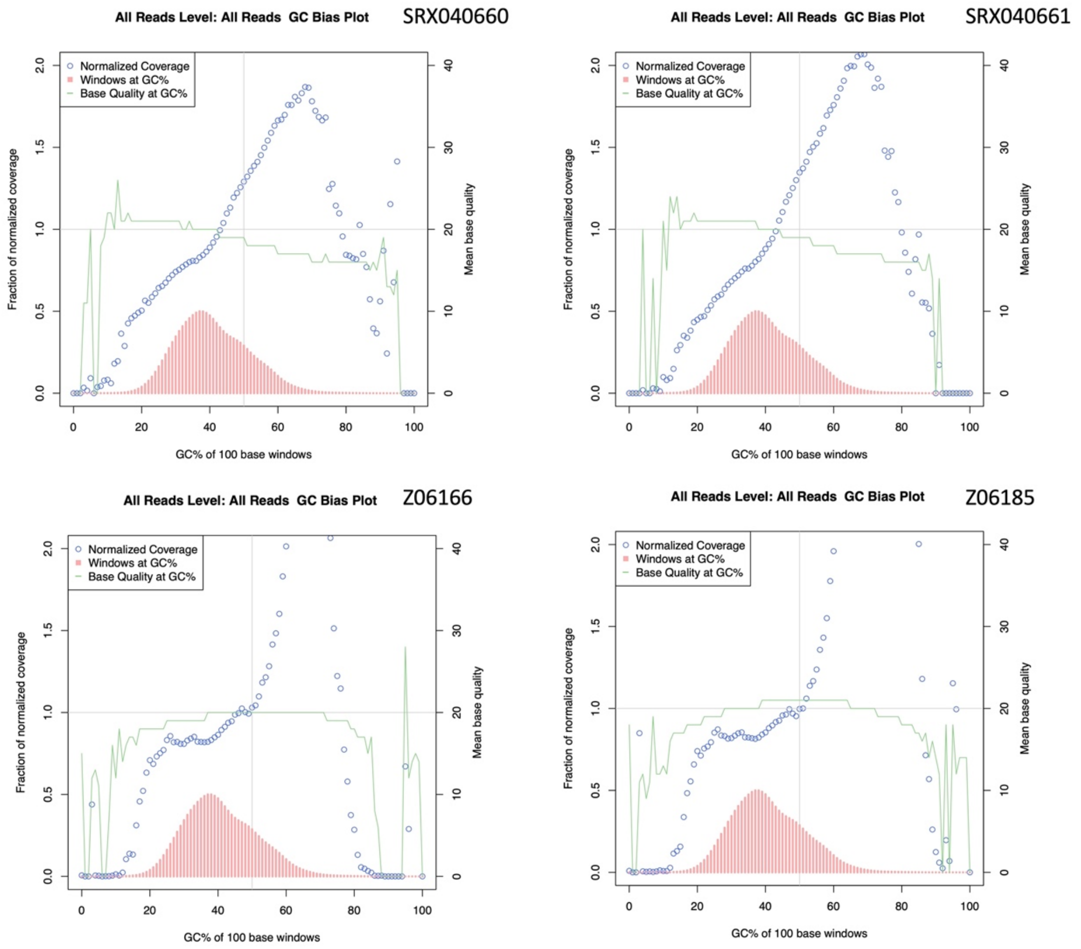

D

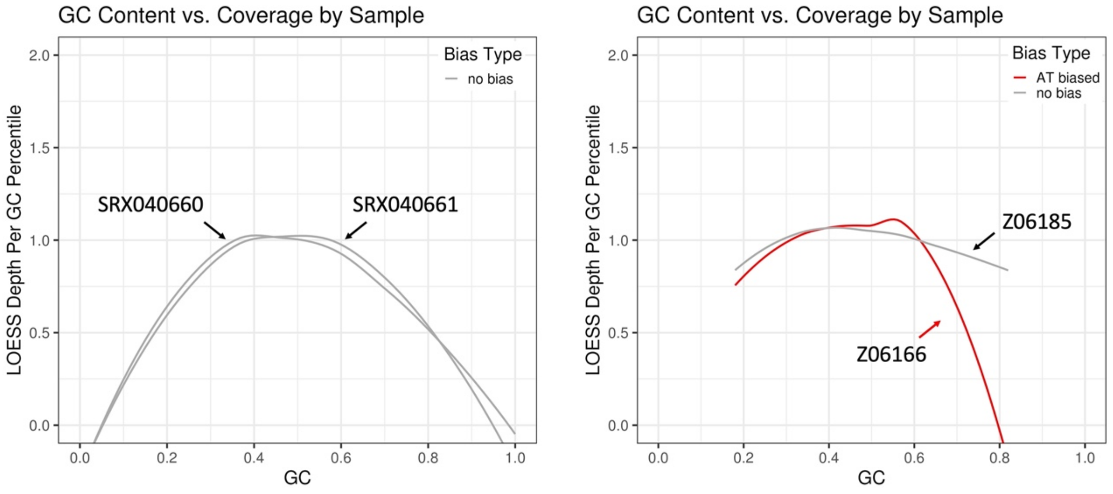

Supplementary Figure 3. GC bias profile plots on simulated hybridization capture sequencing data from published sources and real clinical cell line samples by (A) deepTools, (B) Qualimap, (C) Picard, and (D) panelGC.

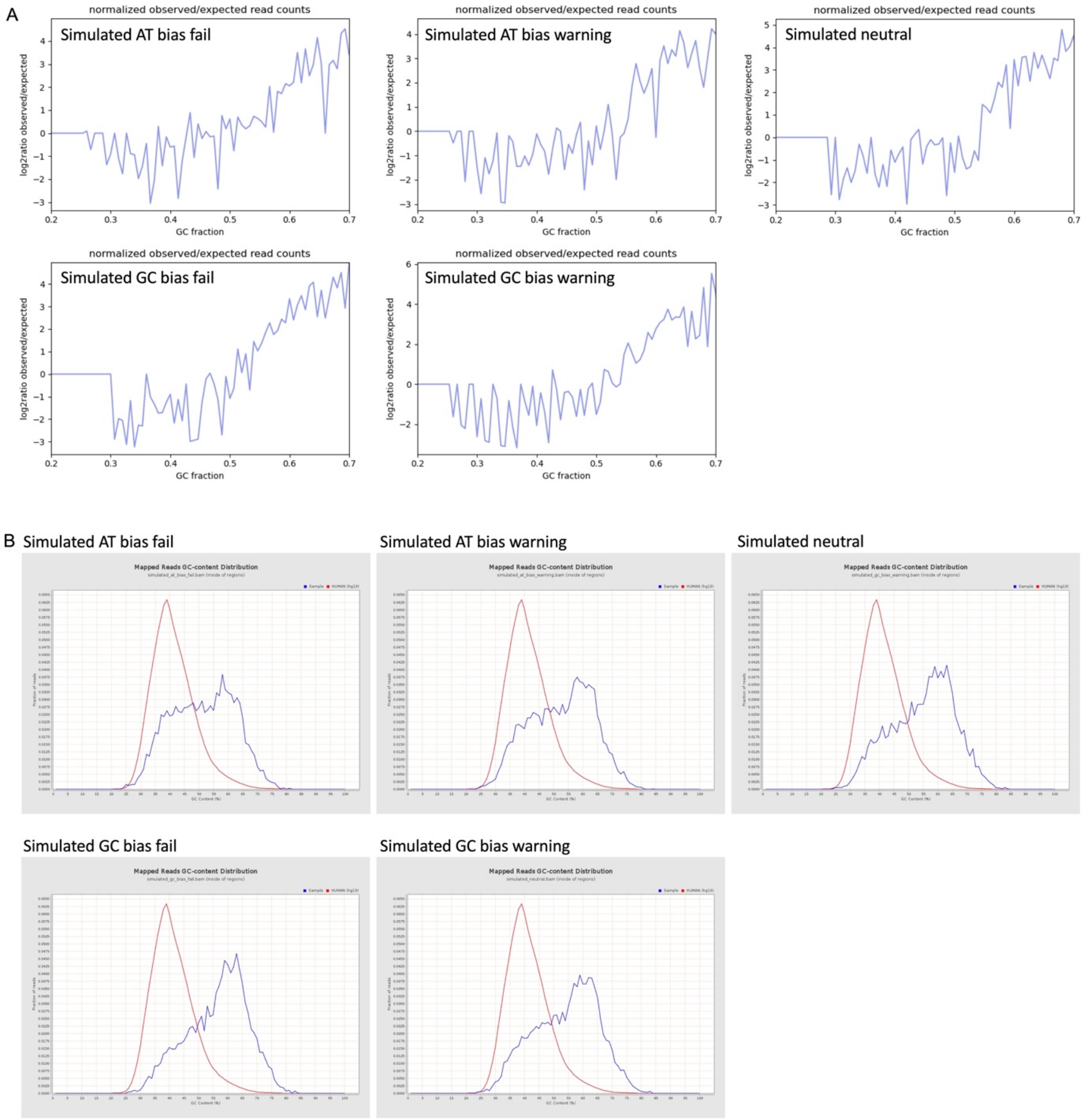
A
normalized observed/expected read counts
Simulated AT bias fail
Simulated AT bias warning
Simulated neutral
Simulated GC bias fail
Simulated GC bias warning
log2ratio observed/expected
GC fraction
B
Simulated AT bias fail
Simulated AT bias warning
Simulated neutral
Simulated GC bias fail
Simulated GC bias warning
Mapped Reads GC-content Distribution

C

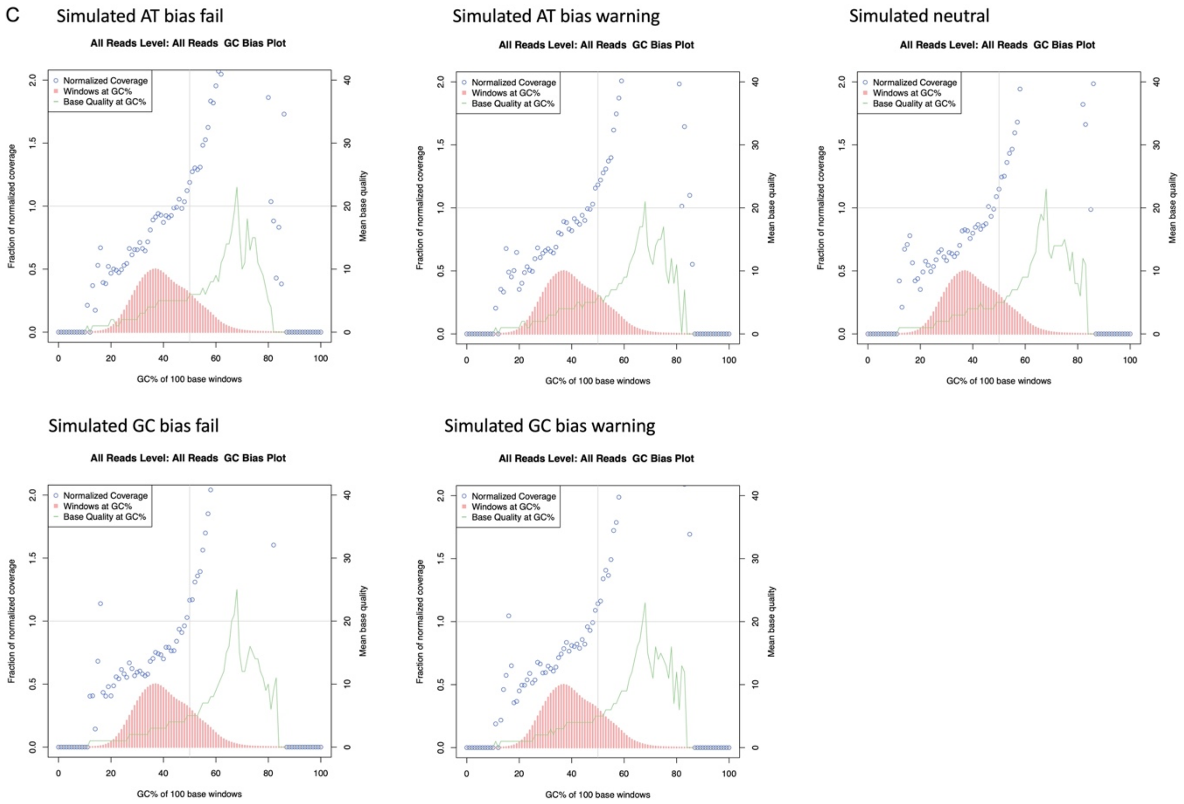

D

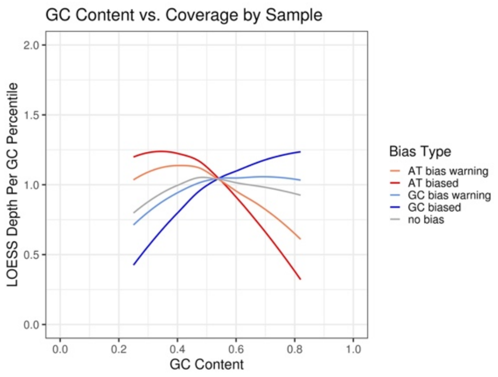

Supplementary Figure 4. GC bias profile plots on simulated panel data with controlled GC and AT biases by (A) deepTools, (B) Qualimap, (C) Picard, and (D) panelGC.
